# Microstructural Investigation of Heat-Treated Ultra-High Performance Concrete for Optimum Production

**DOI:** 10.3390/ma10091106

**Published:** 2017-09-20

**Authors:** Sung-Hoon Kang, Ji-Hyung Lee, Sung-Gul Hong, Juhyuk Moon

**Affiliations:** 1 Department of Architecture & Architectural Engineering, Seoul National University, 1 Gwanak-ro, Gwanak-gu, Seoul 08826, Korea; medesis@snu.ac.kr (S.-H.K.); cb415@snu.ac.kr (J.-H.L.); 2 Department of Civil and Environmental Engineering, National University of Singapore, 1 Engineering Drive 2, Singapore 117576, Singapore

**Keywords:** ultra-high performance concrete, microstructure, pozzolanic reaction, heat-treatment, portlandite

## Abstract

For optimum production of ultra-high performance concrete (UHPC), the material and microstructural properties of UHPC cured under various heat treatment (HT) conditions are studied. The effects of HT temperature and duration on the hydration reaction, microstructure, and mechanical properties of UHPC are investigated. Increasing HT temperature accelerates both cement hydration and pozzolanic reaction, but the latter is more significantly affected. This accelerated pozzolanic reaction in UHPC clearly enhances compressive strength. However, strength after the HT becomes stable as most of the hydration finishes during the HT period. Particularly, it was concluded that the mechanical benefit of the increased temperature and duration on the 28 day-strength is not noticeable when the HT temperature is above 60 °C (with a 48 h duration) or the HT duration is longer than 12 h (with 90 °C temperature). On the other hand, even with a minimal HT condition such as 1 day at 60 °C or 12 h at 90 °C, outstanding compressive strength of 179 MPa and flexural tensile strength of 49 MPa are achieved at 28 days. Microstructural investigation conducted herein suggests that portlandite content can be a good indicator for the mechanical performance of UHPC regardless of its HT curing conditions. These findings can contribute to reducing manufacturing energy consumption, cost, and environmental impact in the production of UHPC and be helpful for practitioners to better understand the effect of HT on UHPC and optimize its production.

## 1. Introduction

One of the advantages of ultra-high performance concrete (UHPC) is outstanding mechanical performance. This includes ultra-high compressive strength (150–250 MPa), non-brittleness, and good load-carrying capacity under tension by incorporating fibers [1,2,3,4]. In order to obtain ultra-high strength at an early stage (<7 days), this composite material is usually subjected to heat-treatment (HT) right after demolding at 12–48 h during the curing stage [5,6]. The standard HT condition for UHPC production is 90 °C of steam curing for 48 h [2,3,7,8]. The increase in curing temperature is known to promote a pozzolanic reaction among silica fume (SF), portlandite, and water [6,9]. This enables the concrete to achieve high early strength that is useful in precast concrete production [8].

However, the required standard HT conditions (temperature and duration) for UHPC is much more demanding compared to the steam curing conditions of normal precast concretes (below 65 °C for a few hours) [8]. The longer steam curing at a higher temperature (i.e., 90 °C) for UHPC can increase manufacturing costs and energy consumption [7,10]. More importantly, special equipment is necessary to maintain 90 °C for several days. Thus, UHPC cannot be readily manufactured using the above mentioned standard HT conditions in precast factories that are only equipped with general concrete precast facilities. This is an additional factor that hinders the widespread use of UHPC.

High material costs, high cement content (around 1000 kg/m^3^), and high temperature curing are all limiting factors for the broader application of UHPC [11,12,13]. As a solution, previous studies have suggested replacing expensive components (such as cement and SF) with cheaper and eco-friendly materials such as limestone, calcined clay, fly-ash, or ground-granulated blast-furnace slag [11,12,14,15,16]. However, cost-effective and less-energy intensive curing methods of UHPC have not been sufficiently studied. Even so, several clues have been found in previous studies of efficient HT conditions for UHPC. For instance, there was no noticeable loss in the mechanical performance of UHPC when HT temperature or duration were reduced [6,17]. Although it was exposed at 65 °C for only one day, portlandite was significantly consumed by the pozzolanic reaction, and the resulting microstructure was densified. In addition, the change of total porosity was more significant in the temperature range of 20–65 °C than in a range of 65–90 °C [8]. In the study by Heinz et al., only a slight strength reduction in UHPC was observed although the applied HT duration (at 90 °C) was greatly reduced from 48 h to 8 h [17]. These previous studies show the possibility of efficient UHPC production by optimizing HT temperature or duration, which will allow reductions in energy consumption and CO_2_ emission from the HT process. 

Although the above-mentioned possibilities have been reported to some extent, comprehensive studies on material characteristics and mechanical properties that can provide the scientific foundation for mitigating the HT conditions of UHPC are still lacking. For instance, the mechanical properties based on HT temperature and duration have only been investigated for early-age compressive strength [18]. The effects of HT temperature on hydration and the microstructural characteristics of UHPC have been studied only at temperatures of 90–450 °C which is not practical [8,9,17,19,20]. In our study, in an effort to make UHPC more cost-effective and environmentally friendly, the effects of mitigated HT conditions on the mechanical properties, hydration reaction, and microstructure of UHPC were systematically investigated. A total of ten UHPC samples cured under selected HT conditions were prepared, and their compressive and flexural strengths were measured at different curing ages. In addition, various microstructural characteristics were investigated by using hydration heat tests, X-ray diffraction (XRD), thermogravimetric analysis (TGA), and mercury intrusion porosimetry (MIP)-based pore structure analysis. Based on extensively conducted experiments, the effectiveness of HT temperature and duration on UHPC performance was discussed.

## 2. Experimental Method

### 2.1. Preparation of Ultra-High Performance Concrete with Various Heat-Hreatment Conditions

**Figure 1:**
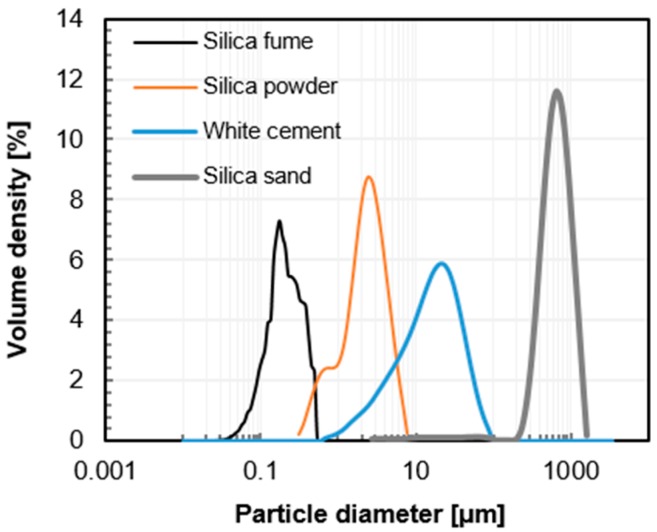
Particle size distribution of raw materials measured by an image analysis method for silica fume and a laser diffraction method for silica powder, white cement, and silica sand.

The mix proportion of UHPC used in this study is presented in Table 1. The dry mixtures were prepared by homogenously blending white Portland cement (Union cement, Seoul, Korea), silica fume (Grade 940U, Elkem, Norway), silica powder, and silica sand using a 5-liter Hobart mixer. The mixing water, composed of tap water and superplasticizer (polycarboxylate-ether type), was added to the dry mixture and mixed until it changed to a liquid state. Finally, the steel fibers (Φ0.2 mm × 13 mm, tensile strength > 2500 MPa) were mixed with the liquid-state concrete and homogeneously dispersed in the mixer. A more detailed mixing procedure is described in our previous study [21]. Table 2 shows the chemical composition of raw materials measured by X-ray fluorescence analysis using XRF-1700 (Shimadzu, Tokyo, Japan). The particle size distributions of the raw materials are shown in Figure 1. The distribution of SF was determined by an image-processing method using a scanning electron microscope (JSM-7800F Prime, JEOL Ltd., Tokyo, Japan), and the others were analyzed by a laser diffraction method (Mastersizer 3000, Malvern Instruments, Malvern, UK) [22,23]. Figure 1 shows that the maximum aggregate size is limited to about 1 mm and the particle size of the dry materials is evenly distributed, to enhance the homogeneity and compactness of UHPC [9]. 

**Figure 2:**
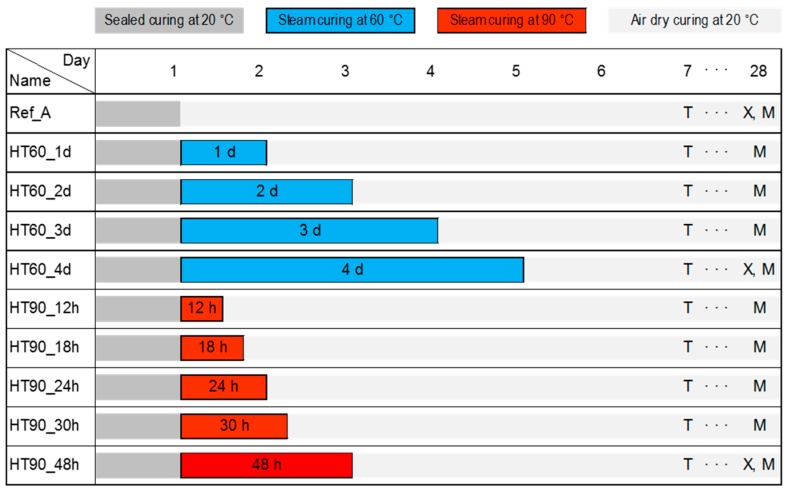
Curing and testing program of UHPC samples (T, X, and M denote the test days for Thermogravimetric Analysis (TGA), X-ray Diffraction (XRD), and mercury intrusion porosimetry (MIP), respectively).

Each fresh sample of UHPC containing steel fibers was immediately sealed after it was poured into a prepared mold. After 24 h, all the samples were demolded, and then 10 different curing programs, shown in Figure 2, were applied in three different curing chambers. Ref_A is a sample directly exposed to dry air (20 °C and RH 60%) after removing the seal. In the sample name, HT60 or HT90 means target HT temperature and applied HT duration in days (d) or hours (h). For instance, HT90_48h is the sample subjected to the standard HT (90 °C and RH 95%) for 48 h. 

### 2.2. Experimental Methods

#### 2.2.1. Isothermal Calorimetry

Hydration heat tests were performed using an isothermal calorimeter (TAM Air, TA Instruments, New Castle, DE, USA), to investigate the hydration reaction of the UHPC. The heat flow and cumulative heat of 15 g of paste were measured by using the curing temperature, as presented in Figure 2. In this measurement, silica sand and steel fiber were excluded since they are inert materials in UHPC formulation. To closely simulate the planned curing program, the temperature of the calorimeter should be quickly increased from 20 °C to 60 °C or 90 °C. However, since it is technically impossible, the alternative method below was applied for measuring the hydration heat of samples cured at a high temperature. Firstly, each sample was cured at 20 °C for 24 h before the HT, and then the hydration heat during the designated HT period was measured by inserting the sample (cured for 24 h) into the calorimeter that had reached the target HT temperature (60 °C or 90 °C). 

#### 2.2.2. Strength

The compressive strength test was carried out according to ASTM C109 [24]. At 1, 3, 7, 28, and 91 days, the strength of the cubic specimens (50 mm × 50 mm × 50 mm) was measured using a universal testing machine; the average and standard deviation of the three specimens are shown in Figure 4. To determine flexural tensile strength, three prismatic specimens (40 mm × 40 mm × 160 mm) per sample were tested at 28 days, according to a three-point bending test method [25].

#### 2.2.3. X-ray Diffraction (XRD) and Thermogravimetric Analysis (TGA)

XRD analysis was performed to investigate the mineralogical characteristics of UHPC with different HT conditions. At 28 days, the paste samples excluding sand and fiber, were crushed and ground, and then the obtained powder was placed in a holder for analysis. The prepared samples were scanned by Miniflex (Rigaku, Tokyo, Japan) with a step size of 0.0033° (2 θ). The crystalline phases were identified by the method described in previous studies [22,23,26]. Thermogravimetric analysis (TGA) was performed to quantitatively computing the portlandite content of all samples. At 7 days of curing, approximately 10 mg of the sample was tested in a nitrogen atmosphere using a Q500 TGA (TA Instrument, New Castle, DE, USA). The sample was heated from 50 °C to 1000 °C with a ramp rate of 10 °C/min. After the TGA, the portlandite content was calculated by the weight loss of water around 400–500 °C using a tangential method [12,27]. For normalization, the portlandite content was rescaled by the solid fraction of the sample that was obtained from the remaining weight at 500 °C [27,28].

#### 2.2.4. Pore Size Distribution

MIP (AutoPore IV 9500, Micromeritics, Norcross, GA, USA) was performed to investigate the porosity and pore size distribution of the UHPC. The UHPC that had been cured for 28 days was cut into small pieces (about 5 mm cubes), and then the hydration reaction of the cubes was stopped by following a procedure [29]. The set parameters for MIP were 485 erg/cm^2^ for surface tension and 130° for the contact angle of mercury. 

## 3. Results

### 3.1. Heat of Hydration

**Figure 3:**
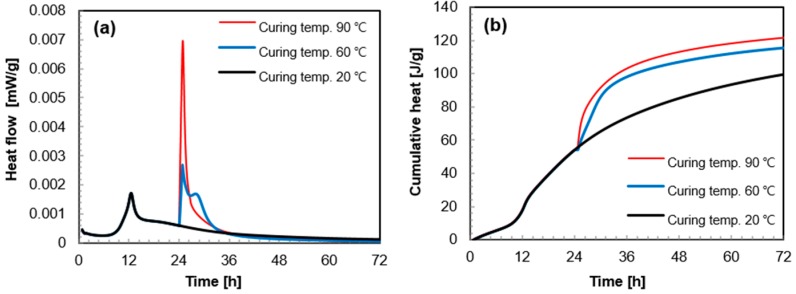
Heat flow (**a**) and cumulative heat (**b**) of UHPC cured under 20 °C, 60 °C, and 90 °C (the elevated temperature curing at 60 °C or 90 °C started from 24 h).

The heat flow and cumulative heat released by a unit weight of paste are shown in Figure 3a,b, respectively. Before the start of HT, the first peak of hydration heat was formed around 12 h in the Ref_A sample, and thereafter the heat flow gradually decelerated. The hydration heat did not increase after the formation of the first peak (see black line in Figure 3a). However, as soon as the curing temperature increased at 24 h from 20 °C to 60 °C or 90 °C, the hydration heat sharply increased and the second peak was formed. The higher the temperature, the higher the peak height, i.e., the peak height at 90 °C is twice as high than that at 60 °C. Because of this additional peak formation, the cumulative heat also increased substantially after the start of the HT (Figure 3b). However, the increase in the hydration heat by the HT was effective only during the initial 12 h of the HT period. Thereafter, the heat flow of the sample with HT steadily decreased as the Ref_A sample does (Figure 3a), and thus the rate of cumulative heat also stabilized (Figure 3b). 

### 3.2. Mechanical Properties

**Figure 4:**
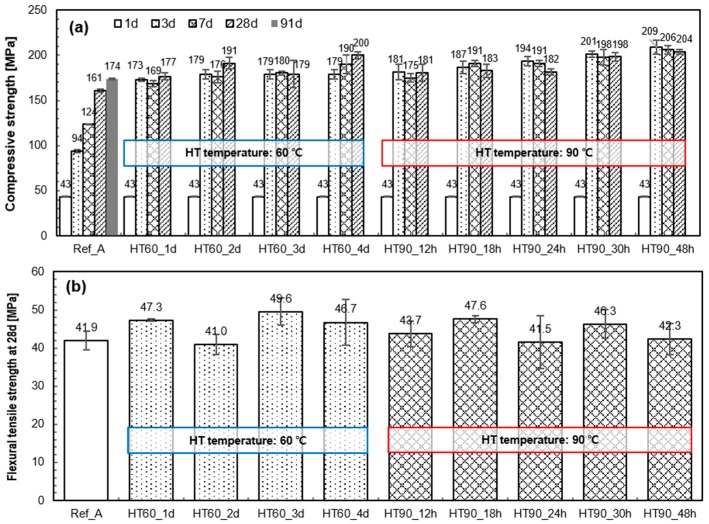
Compressive strength development (**a**) and flexural tensile strength at 28 days (**b**) of UHPC with different HT conditions.

Figure 4a,b shows the compressive strength of the samples with different curing periods and the flexural tensile strength at 28 days, respectively. At 28 days, all samples satisfied the strength requirement for UHPC (i.e., compressive strength > 150 MPa). In the case of the sample without HT (Ref_A), the compressive strength constantly increased with curing age, and a high strength of 174 MPa was achieved at 91 days. In addition, all heat-treated samples exhibited outstanding strength (189 ± 20 MPa) after 7 days. However, the tendency of compressive strength development between 7–28 days was somewhat different based on the HT temperature. After HT, the samples that experienced 60 °C tended to maintain or increase their strength until 28 days, whereas those that experienced 90 °C tended to maintain or decrease their strength. Meanwhile, the flexural tensile strength was not influenced by the HT temperature and duration. All samples exhibited a flexural tensile strength of 40–50 MPa at 28 days, although there is no clear tendency as seen in Figure 4b. 

### 3.3. Mineralogical Analysis

**Figure 5:**
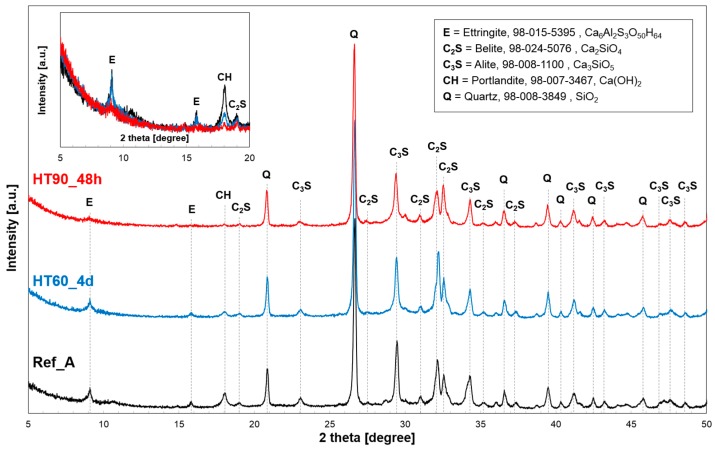
XRD patterns of UHPC at 28 days: E = ettringite, C_2_S = belite, C_3_S = alite, CH = portlandite, Q = quartz.

Figure 5 shows the results of XRD analysis of three samples cured for 28 days. At the main crystalline phases in UHPC, inert quartz, ettringite, unreacted cement clinkers, and portlandite are identified. The quartz peak is detected from crystalline SiO_2_ contained in silica powder (i.e., not from the silica sand which is excluded for the XRD test), and its intensity does not noticeably change in different curing conditions. This is because, unlike SF, silica powder is known to act as an inert filler in UHPC unless it is exposed to 150 °C or higher temperature [6,19]. In the case of the detected ettringite mineral, the HT of 60 °C did not change the peak intensity, whereas the peak almost disappeared at a HT of 90 °C. It is known that when concrete is exposed to temperatures above 70 °C, ettringite in the concrete starts to decompose [30]. In this case, expansion or damage due to delayed ettringite formation (DEF) might be considered in the later stages. Although the risk of the DEF in the case of UHPC has not been fully explained yet, the effect of DEF can be neglected in UHPC because there is no available water for later ettringite formation, especially under the HT [8]. 

UHPC contains a large quantity of unreacted cement clinkers despite the HT [12,14,31,32]. This is primarily because there is an insufficient amount of water for the complete hydration of the cement [7,33]. It is also confirmed in our study that HT promotes cement hydration at higher curing temperatures (see reduced peak intensities of alite and belite in Figure 5). On the other hand, the HT greatly promoted the portlandite consumption by the pozzolanic reaction in the UHPC. The intensity of the portlandite peak significantly decreased at higher temperature curing, and the peak almost disappeared after the HT at 90 °C for 48 h.

**Figure 6:**
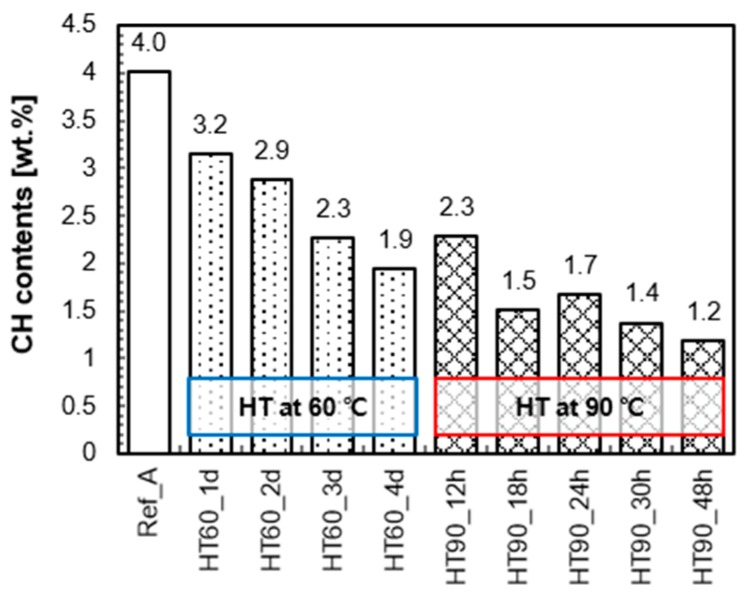
Portlandite contents in UHPC at 7 days measured by TGA.

The effect of HT temperature and duration on the pozzolanic reaction of UHPC was quantitatively investigated by TGA. Figure 6 shows the calculated portlandite contents of all samples measured at 7 days. Increasing the curing temperature and duration were both effective in accelerating the pozzolanic reaction. The decrease in the portlandite contents means additional formation of C-S-H, which improves the mechanical performance by filling voids in the UHPC [11]. The portlandite content in HT60_1d (3.2%) is 0.8% lower than that in Ref_A (4.0%). An additional 3 days of HT at 60 °C also reduced the portlandite content (1.9% in HT60_4d). Moreover, the HT at 90 °C for 12–18 h significantly reduced the portlandite contents from 4.0% (Ref_A) to 2.3% (HT90_12h) or 1.5% (HT90_18h), which is a similar or lower level compared to the case of HT at 60 °C for 3–4 days, respectively. However, HT for longer than 18 h at 90 °C did not result in a higher degree of pozzolanic reaction. 

### 3.4. Pore Structure Analysis

**Figure 7:**
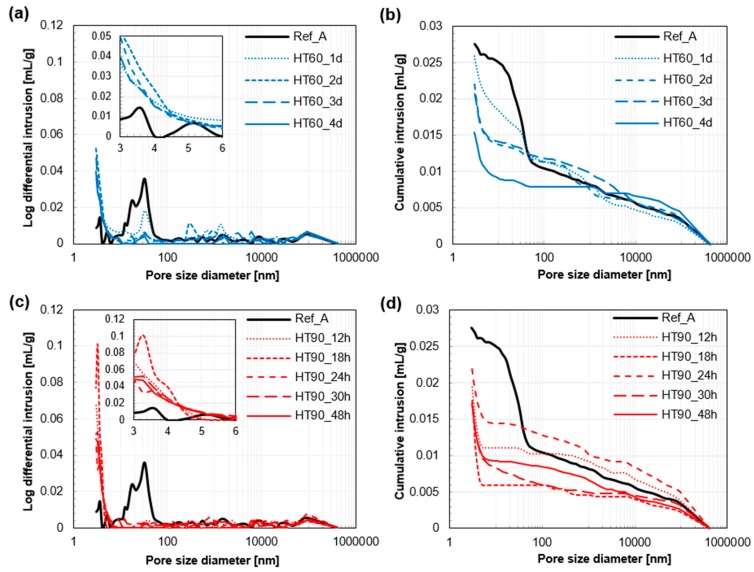
Pore size distribution of UHPC at 28 days: Log differential intrusions (**a**) and cumulative pore volume (**b**) of Ref_A and HT_60 series. Log differential intrusions (**c**) and cumulative pore volume (**d**) of Ref_A and HT_90 series.

Pore size distributions of the samples at 28 days are shown in Figure 7. All samples have almost the same shape and size of pore peaks at 20 μm, which is formed by entrapped or entrained air bubbles [34]. This also confirms that the conducted MIP experiment is reliable. Except for the large pores, the pore structure of UHPC was completely changed by the HT [9,35]. In particular, the capillary voids broadly formed at 10–100 nm were remarkably reduced due to the HT. In the case of the HT_60 series, the pore volume at this range decreased with increasing HT duration. However, those in the HT_90 series were significantly reduced by applying only 12 h of HT, and did not show a certain trend of change depending on the duration of the HT. In addition, a significant number of pores smaller than 5 nm were newly generated after the HT. In summary, HT made the pore structure of UHPC considerably finer, within the range of 100 nm or less, and contributed to lowering the total porosity. 

**Figure 8:**
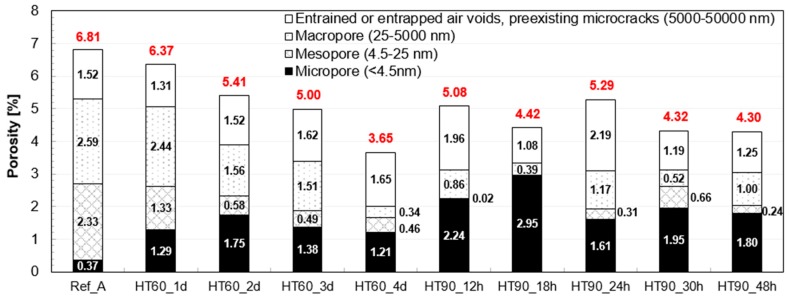
Pore volume distributions of UHPC at 28 days (Red numbers on the bars indicate the total porosity).

Figure 8 shows the porosity of the samples, which is divided into four ranges based on pore diameter [22,23]. The total porosity was reduced by the HT at 60 °C, and this became more prominent as it cured longer. Only pores below 5 μm contributed to this reduction, and as mentioned previously, the volume of the entrained or entrapped air voids did not vary according to the HT. In all heat-treated samples, the volumes of micropores and mesopores significantly changed to increase and decrease, respectively. The HT at 90 °C was also effective in reducing the total porosity, but its long duration did not always lead to a lower total porosity. In other words, there was no significant decrease in the total porosity when the HT duration was longer than 18 h. Compared to the HT at 60 °C, the HT at 90 °C made the pore structure much finer especially below 25 nm. Thus, the fraction of micropore volume of the HT_90 series is particularly high.

## 4. Discussion

### 4.1. Pozzolanic Reaction of UHPC Subject to Heat Treatment

Heat treatment significantly promotes the pozzolanic reaction among portlandite, water, and amorphous silica [9,17,22,23]. Heat treatment also accelerates the hydration of clinker phases (Figure 5). Since portlandite is formed as a result of the hydration of clinker phases such as alite and belite [34], the HT should promote both portlandite production from the accelerated cement hydration, and portlandite consumption from the pozzolanic reaction. However, as can be confirmed by the portlandite peaks in Figure 5, the reaction to consume portlandite is more significant than that to produce it; thus, the amount of portlandite identified was reduced as the HT temperature and duration increased. Meanwhile, SF is a major source of silica for the pozzolanic reaction, and its reactivity is also significantly improved by the HT [9,31]. Previously, it was reported that almost half of the SF in UHPC (selected ratio of SF to cement: 16%) is reacted under the standard HT condition (at 90 °C for 48 h), and this also means that the other half simply stays as a physical filler [36]. 

In UHPC, the pozzolanic reaction progresses markedly after the second day of curing [31]. The HT initiated at this time, further accelerates the water consumption [35]. In particular, this reaction clearly accelerated during the initial 12 h of the HT period (Figure 3), simultaneously making the UHPC denser due to the additionally formed amorphous hydration products. However, after this (>12 h), the hydration reaction begins to decrease because of the lack of available water or insufficient space to form the hydration products [37]. As a result, the pozzolanic reaction was effectively accelerated for 12 h after the initiation of the HT.

**Figure 9:**
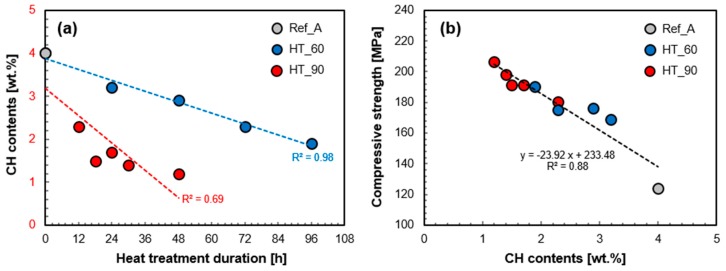
Portlandite content (at 7 days) as a function of HT temperature and duration (**a**); and found relationship between portlandite content and compressive strength at 7 days (**b**).

Figure 9a quantitatively shows the effect of HT temperature and duration on the portlandite content and the pozzolanic reaction. It was confirmed that the reaction is promoted by increasing HT temperature and duration. In the case of the HT at 90 °C, the rate of portlandite consumption per HT hour is 2.5 times faster compared to the HT at 60 °C. This indicates that if the HT temperature is increased from 60 °C to 90 °C, the pozzolanic reaction can be greatly accelerated for a shorter period of time. On the other hand, if the temperature has to be reduced from 90 °C to 60 °C (e.g., due to curing equipment limitations), the duration should be longer to assure a pozzolanic reaction of the same degree. 

The pozzolanic reaction plays a crucial role in early strength development of the UHPC [7,31,38]. Thus, the accelerated pozzolanic reaction by HT is a major factor for enhancing compressive strength at an early stage such as 7 days [6]. This is clearly demonstrated in Figure 9b; there is a linear correlation between portlandite content and compressive strength at 7 days. This result supports the fact that the initial strength increased as HT temperature and duration increased, is mostly due to the acceleration of the pozzolanic reaction. Based on this correlation, the ultimate strength of about 233 MPa can be predicted if the pozzolanic reaction is entirely progressed and all portlandite is consumed. On the other hand, a drop of compressive strength of about 24 MPa can be expected when the portlandite content is increased by 1%. Thus, another important implication from Figure 9b is that engineers or researchers may predict the long-term strength of a UHPC structure accurately at any time within its life-span, by measuring portlandite content. Additionally, other factors which can affect long-term strength such as microcracks due to shrinkage or deterioration of durability due to ion penetration can be safely ignored in UHPC. It is previously known that both shrinkage and chloride permeability of UHPC after HT are negligibly small [1,2,3,39].

### 4.2. Benefits of Heat-Treatment on the Mechanical Properties of UHPC

**Figure 10:**
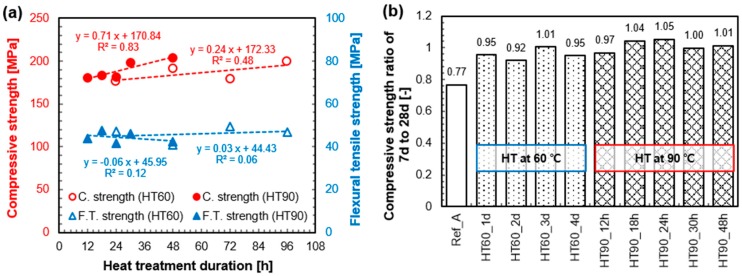
28 day-strength of UHPC as a function of HT temperature and duration (**a**); and compressive strength ratio of 7 days to 28 days (**b**).

Figure 10a shows how the applied HT condition enhances compressive strength and flexural tensile strength of UHPC at 28 days. The compressive strength increased as a function of the temperature and duration. On average, its rate was 0.24 MPa/h for HT at 60 °C, and 0.71 MPa/h for HT at 90 °C. The HT at 90 °C is more effective (about 3 times) than HT at 60 °C. However, the samples with minimal HT conditions, such as HT60_1d or HT90_12h also show excellent compressive strength (179 ± 2 MPa). This observation may raise the question as to why we apply higher temperature or longer duration to further increase the compressive strength of UHPC. Moreover, this demanding HT condition did not make a positive contribution to the increase in flexural tensile strength (Figure 10a). Along with compressive strength, the superior flexural or tensile strength is another advantage of UHPC because it provides a minimum reinforcement in the concrete [1]. It has been also previously reported that the distribution and orientation of included fibers will have a decisive effect on its performance, rather than applied HT conditions [40,41,42].

The strength ratios of 7 days compared to 28 days are presented in Figure 10b. It shows the strength changes up to 28 days after the end of HT. When UHPC was heat-treated for longer than 18 h at 90 °C, the strength tended to decrease between 7 days and 28 days. Two factors can contribute to this reduction. First, due to the accelerated pozzolanic reaction during the HT period, most of water in UHPC is consumed and in turn the microstructure becomes dense. Therefore, the pozzolanic reaction after the HT becomes very weak. Along with this, when the UHPC is exposed to a high temperature such as 90 °C, the strength is thereafter reduced until about the 28th day because of a relaxation phenomenon [8]. On the other hand, the strength of samples treated at 60 °C did not decrease after the HT, but rather slightly increased owing to a relatively large amount of available water for the pozzolanic reaction. These samples exhibited at least 90% of the 28 day-strength at 7 days. Although it is less than 100%, such a fast rate of strength development is beneficial considering precast manufacturing conditions which requires rapid strength development for transportation and installation at an early stage. 

## 5. Conclusions

The hydration characteristics and mechanical properties of UHPC with various HT temperatures and durations were investigated. The following conclusions are drawn from the results of this study. 

During the HT period, the hydration reaction was accelerated by an increasing curing temperature. Although both the first (cement hydration) and second (pozzolanic reaction) reactions could be promoted by HT, the second reaction was more obviously accelerated than the first reaction. As the HT duration increases, the pozzolanic reaction was also promoted. These increases in temperature and duration made the pore structure of UHPC finer (particularly in the range below 100 nm), which contributed to improving its mechanical performance. Therefore, a linear correlation between remaining portlandite contents and compressive strength could be formulated. 

Heat treatment at high temperatures (i.e., 90 °C) ensured outstanding strength after the HT. In this case, however, there was no increase in the strength between 7 days and 28 days, but rather a slight decrease was observed. However, after the HT at low temperatures (i.e., 60 °C), the strength tended to continue to increase until 28 days. Consequently, the HT at 90 °C and 60 °C did not show a noticeable difference in the 28 day-compressive strength. Moreover, the flexural tensile strengths at 28 days of all samples were similar regardless of curing conditions. 

Increasing the HT temperature and duration is certainly an effective way to improve the compressive strength of UHPC at the point immediately after HT. However, its efficiency can decrease if performance at later stages is considered. More importantly, the mitigated HT conditions that has been suggested herein, can sufficiently accelerate strength development and ensure outstanding mechanical performance for UHPC. Thus, it can be reasonably used as a more economical and practical way for producing precast UHPC.

## Figures and Tables

**Table 1:** Mix proportion of prepared ultra-high performance concrete (UHPC) (wt % of cement).

Cement	Silica Fume	Silica Powder	Silica Sand	Water	Superplasticizer ^1^	Steel Fiber [vol %] ^2^
1	0.25	0.35	1.1	0.253	0.012	2

^1^ Solid content; ^2^ Volume ratio of UHPC.

**Table 2:** Oxide compositions of raw materials used (wt %).

Chemical Composition	SiO_2_	Al_2_O_3_	Fe_2_O_3_	MgO	CaO	K_2_O	SO_3_	SrO	LOI ^1^	Total
White cement	21.4	4.63	0.358	1.27	65.8	0.13	2.89	0.076	3.4	99.95
Silica fume	96.90	0.29	0.15	0.18	1.54	0.64	-	-	0.02	99.72
Silica powder	97.70	0.49	0.05	0.21	1.37	0.02	-	-	0.02	99.86

^1^ Loss of ignition.

## References

[1] Association Française de Génie Civil (AFGC) (2013). Ultra High Performance Fibre-Reinforced Concrete-Recommendations.

[2] Japan Society of Civil Engineers (JSCE) (2004). Recommendations for Design and Construction of Ultra High-Strength Fiber-Reinforced Concrete Structures-Draft.

[3] Korea Concrete Institute (KCI) (2012). Design Recommendations for K-UHPC.

[4] Toutlemonde F., Delort M. (2016). The newly enforced french standard for uhpfrc specification, performance, production, and conformity. Ultra-High Performance Concrete and High Performance Construction Materials, Proceedings of the 4th International Symposium on Ultra-High Performance Concrete and High Performance Materials, Kassel, Germany, 9–11 March 2016.

[5] National Precast Concrete Association (NPCA) (2013). Ultra High Performance Concrete (UHPC): Guide to Manufacturing Architectural Precast UHPC Elements.

[6] Schachinger I., Hilbig H., Stengel T. (2008). Effect of curing temperature at an early age on the long-term strength development of UHPC. Ultra High Performance Concrete (UHPC), Proceedings of the 2nd International Symposium on Ultra High Performance Concrete, Kassel, Germany, 5–7 March 2008.

[7] Selleng C., Meng B., Fontana P. (2016). Phase composition and strength of thermally treated UHPC. Ultra-High Performance Concrete and High Performance Construction Materials, Proceedings of the 4th International Symposium on Ultra-High Performance Concrete and High Performance Materials, Kassel, Germany, 9–11 March 2016.

[8] Heinz D., Ludwig H.-M. (2004). Heat treatment and the risk of DEF delayed ettringite formation in UHPC. Ultra High Performance Concrete (UHPC), Proceedings of the 1st International Symposium on Ultra-High Performance Concrete Kassel, Germany, 13–15 September 2004.

[9] Richard P., Cheyrezy M. (1995). Composition of reactive powder concretes. Cem. Concr. Res..

[10] Yazıcı H., Deniz E., Baradan B. (2013). The effect of autoclave pressure, temperature and duration time on mechanical properties of reactive powder concrete. Constr. Build. Mater..

[11] Yu R., Spiesz P., Brouwers H. (2015). Development of an eco-friendly ultra-high performance concrete (UHPC) with efficient cement and mineral admixtures uses. Cem. Concr. Compos..

[12] Huang W., Kazemi-Kamyab H., Sun W., Scrivener K. (2017). Effect of cement substitution by limestone on the hydration and microstructural development of ultra-high performance concrete (UHPC). Cem. Concr. Compos..

[13] Park J.-J., Yoo D.-Y., Park G.-J., Kim S.-W. (2017). Feasibility of Reducing the Fiber Content in Ultra-High-Performance Fiber-Reinforced Concrete under Flexure. Materials.

[14] Huang W., Kazemi-Kamyab H., Sun W., Scrivener K. (2017). Effect of replacement of silica fume with calcined clay on the hydration and microstructural development of eco-UHPFRC. Mater. Des..

[15] Yazıcı H. (2007). The effect of curing conditions on compressive strength of ultra high strength concrete with high volume mineral admixtures. Build. Environ..

[16] Afroughsabet V., Biolzi L., Ozbakkaloglu T. (2016). High-performance fiber-reinforced concrete: A review. J. Mater. Sci..

[17] Heinz D., Urbonas L., Gerlicher T. (2012). Effect of heat treatment method on the properties of UHPC. Ultra-High Performance Concrete and Nanotechnology in Construction, Proceedings of the 3rd International Symposium on Ultra High Performance Concrete and Nanotechnology for High Performance Construction Materials, Kassel, Germany, 7–9 March 2012.

[18] Park J.-S., Kim Y.J., Cho J.-R., Jeon S.-J. (2015). Early-age strength of ultra-high performance concrete in various curing conditions. Materials.

[19] Zanni H., Cheyrezy M., Maret V., Philippot S., Nieto P. (1996). Investigation of hydration and pozzolanic reaction in reactive powder concrete (RPC) using 29 Si NMR. Cem. Concr. Res..

[20] Reda M., Shrive N., Gillott J. (1999). Microstructural investigation of innovative UHPC. Cem. Concr. Res..

[21] Kang S.-H., Hong S.-G., Moon J. (2017). Absorption kinetics of superabsorbent polymers (SAP) in various cement-based solutions. Cem. Concr. Res..

[22] Kwon Y.-H., Kang S.-H., Hong S.-G., Moon J. (2017). Acceleration of intended pozzolanic reaction under initial thermal treatment for developing cementless fly ash based mortar. Materials.

[23] Kwon Y.-H., Kang S.-H., Hong S.-G., Moon J. (2017). Intensified pozzolanic reaction on kaolinite clay-based mortar. Appl. Sci..

[24] American Society for Testing and Materials (ASTM) (2013). Standard Test Method for Compressive Strength of Hydraulic Cement Mortars (Using 2-in or [50-mm] Cube Specimens).

[25] International Organization for Standardization (ISO) (2009). Cement—Test Methods—Determination of Strength.

[26] Belsky A., Hellenbrandt M., Karen V.L., Luksch P. (2002). New developments in the Inorganic Crystal Structure Database (ICSD): Accessibility in support of materials research and design. Acta Crystallogr. Sect. B.

[27] Scrivener K., Snellings R., Lothenbach B. (2016). A Practical Guide to Microstructural Analysis of Cementitious Materials.

[28] Schöler A., Lothenbach B., Winnefeld F., Zajac M. (2015). Hydration of quaternary Portland cement blends containing blast-furnace slag, siliceous fly ash and limestone powder. Cem. Concr. Compos..

[29] Wu Z., Shi C., Khayat K.H. (2016). Influence of silica fume content on microstructure development and bond to steel fiber in ultra-high strength cement-based materials (UHSC). Cem. Concr. Compos..

[30] Taylor H., Famy C., Scrivener K. (2001). Delayed ettringite formation. Cem. Concr. Res..

[31] Korpa A., Kowald T., Trettin R. (2009). Phase development in normal and ultra high performance cementitious systems by quantitative X-ray analysis and thermoanalytical methods. Cem. Concr. Res..

[32] Lee H.-S., Jang H.-O., Cho K.-H. (2016). Evaluation of Bonding Shear Performance of Ultra-High-Performance Concrete with Increase in Delay in Formation of Cold Joints. Materials.

[33] Jensen O.M., Hansen P.F. (2001). Water-entrained cement-based materials: I. Principles and theoretical background. Cem. Concr. Res..

[34] Mehta P.K., Monteiro P.J. (2006). Concrete: Microstructure, Properties and Materials.

[35] Garas V.Y., Kahn L.F., Kurtis K.E. (2009). Short-term tensile creep and shrinkage of ultra-high performance concrete. Cem. Concr. Compos..

[36] Pfeifer C., Moeser B., Weber C., Stark J. Investigations of the pozzolanic reaction of silica fume in Ultra-high performance concrete (UHPC). Proceedings of the International RILEM Conference on Material Science-MATSCI.

[37] Justs J., Wyrzykowski M., Bajare D., Lura P. (2015). Internal curing by superabsorbent polymers in ultra-high performance concrete. Cem. Concr. Res..

[38] Wu Z., Shi C., Khayat K.H., Wan S. (2016). Effects of different nanomaterials on hardening and performance of ultra-high strength concrete (UHSC). Cem. Concr. Compos..

[39] Abbas S., Soliman A.M., Nehdi M.L. (2015). Exploring mechanical and durability properties of ultra-high performance concrete incorporating various steel fiber lengths and dosages. Constr. Build. Mater..

[40] Zhou B., Uchida Y. (2017). Influence of flowability, casting time and formwork geometry on fiber orientation and mechanical properties of UHPFRC. Cem. Concr. Res..

[41] Abrishambaf A., Pimentel M., Nunes S. (2017). Influence of fibre orientation on the tensile behaviour of ultra-high performance fibre reinforced cementitious composites. Cem. Concr. Res..

[42] Choi M.S., Kang S.-T., Lee B.Y., Koh K.-T., Ryu G.-S. (2016). Improvement in predicting the post-cracking tensile behavior of ultra-high performance cementitious composites based on fiber orientation distribution. Materials.

